# Forty-Second Annual Meeting of the Mississippi Valley Association of Dental Surgeons

**Published:** 1886-04

**Authors:** E. G. Betty


### DISCUSSIONS.

### Discussions at the Forty-Second Annual Meeting of the Mississippi Valley Assocaition of Dental Surgeons, March 3d and 4th, 1886.

REPORTED BY E. G. BETTY, D.D.S.

The meeting of this Association was called to order, by Pres. Emminger promptly on time. After the transaction of routine business the following program was presented by the Executive Committee:

1. "Treatment of Pulpless Teeth."—J. W. JAY, Richmond, Indiana.

2. "Some Phenomena Observed in the Use of Antiseptics in Oral Surgery."—H. A. SMITH, Cincinnati, Ohio.

3. "Bridge Work."—J. E. LOW, Chicago, Ill.

4. Clinic, Bridge Work, Artificial Crowns, Herbst's Method, Dental Appliances, etc.

5. Report of a Case of Death by the Lodgment of a Partial Gold Denture in the Bronchial Tubes.—J. W. JAY, Richmond, Indiana.

6. Amalgam Fillings from the Standpoint of To-day.—C. M. WRIGHT, F. A. HUNTER.

Dr. A. G. Rose read a paper on the first subject, and Dr. G. W. Smith one on Peroxide of Hydrogen; Dr. H. A. Smith read his paper, all of which will be found elsewhere in the REGISTER. The two subjects were then discussed together.

*Dr. H. A. Smith.*—I will cite a case or two to elucidate some of the points in my paper which may not appear sufficiently clear. I had under treatment several molars in the same mouth in which there was considerable softening of the dentine, etc., but no pain. In these cases there is a decalcified layer in which germs are usually found. My method is to isolate the teeth with the rubber dam, dry out the cavities thoroughly, apply an antiseptic, renew the drying with warm air current and then introduce a moisture-tight filling; all this is done to kill the micro-organisms. This is then allowed to remain awhile when it is removed, the antiseptic re-applied and the permanent filling put in. In such cases as these, after a longer or shorter lapse of time, the pulp becomes disturbed and finally dies, exhibiting all the putrefactive *sequelle.* Now then, the question arises, how does this occur? Have the micro-organisms been killed, or has their growth and formation merely been retarded for a period? We should be sure that the antiseptic we use is a germicide and kill the germs for all time.

*Dr. H. J. McKellops.*—Dr. Smith, what do you consider to be the best antiseptic?

*Dr. H. A. Smith.*—Well, that is a question. Some say hydrogen peroxide. I think carbolic acid is the best if used full strength, and we can frequently so use it without danger of producing any ill effects. An abscess with a fistulous opening can be cured just as easily with injections of pure water as with hydrogen peroxide, as it is the flushing out in such instances that does the work. Prof. Charles Mayr has given a good exposition of the action of hydrogen peroxide, and he says it acts mechanically in cleansing out the sac and suppurative surfaces by giving off oxygen. I think that is the correct position to take in regard to it.

*Dr. J. S. Cassidy.*—The papers that were read are very fine and suggestive. I have one criticism, however, to make on the papers and as well upon the way we, as dentists and physicians, speak indiscriminately of antiseptics. I greatly prefer the term—germicide. A germicide may be neither antiseptic nor disinfectant. Lister says an antiseptic destroys germs. With me the question is *do* antiseptics destroy germs? I am not sure that they do. The object of the use of an antiseptic is to place a healthy, or fairly healthy tissue in a condition to resist putrefaction. An antiseptic is the lock on the stable door. A solution of hydro-chloride of cocaine when exposed, will grow fungi. Will sylicylic acid destroy that fungus after it has been formed? No, but a small portion of it in the cocaine will prevent the formation of the fungi; it is the lock on the stable door. If the part is putrefied and the germs have formed, a *germicide* is indicated. With us, in our treatment of decayed teeth, all our antiseptics, disinfectants, and germicides, consist of the free use of instruments in the removal of the affected part. Nature's disinfectant is the abundant supply and use of water.

Hydrogen peroxide is not liquid oxygen, but a compound that liberates oxygen. In contact with pus it produces ebullition. The putrid gas in an abscess is oxidized by the oxygen liberated.

Adjourned.

AFTERNOON SESSION.

When the meeting was called to order Dr. J. W. Jay read his paper on the first subject of the programme. (It is printed elsewhere in the REGISTER.) The discussion of the morning was resumed.

*Dr. H. A. Smith.*--I would like to call attention to an experiment to prevent the formation of germs in fermentable fluids. Two sugar solutions are separated by a layer of dry cotton; the upper one is infected and shows the formation of germs, while the lower solution will remain untainted by them.

Our success in the treatment of pulpless teeth depends upon our intelligent use of antiseptics, though we don't know enough about them yet; we have used them clinically and empirically. The question in my mind is, don't the dry cotton of the experiment constitute a good obstacle to the entrance of asceptic germs?

*Dr. H. J. McKellops*—Pulpless teeth give us a great deal of trouble. Where does it come from? Where is it? Is it simply in the canal, at the apex, or the surroundings of the root? *There* is where the trouble is and that is where we must reach. When the canal is opened the gas eecapes, and thus nature relieves itself. If the tooth is treated and filled at once, the trouble will recur. The trouble is in the socket and lining membrane. Time sufficient must be spent in a course of proper treatment to remove the cause, and then the treatment can be successful with some degree of certainty. Another thing about this method or habit of leaving a portion of softened dentine over pulps; there must be something done to destroy the process of destruction or it will recur sooner or later—in three months—six months—a year or a year and a half. Theory is all well enough, but give us something practical, something we can get at, so that we can do our patients some practical good.

*Dr. F. W. Sage.*—Dr. H. A. Smith's paper contains matter that seems to revolutinize all our thoughts and methods on the subject, or rather to ante-date all our established theories. For instance, the pushing of *debris* through the apical foramen. If the germ theory be true, we have received a good deal of light upon treatmetnt that will avoid consequent trouble, as, for instance, the theory of Lister, though his results do not seem to be what they are claimed to be, as others have failed to secure the results he claims can be obtained. Our attention has been called to hydrogen peroxide as a new remedy, whereas it is an old one mentioned in old editions of the United States Dispensatory, though in dentistry the conditions seem to be peculiarly susceptible to display its most beneficial effects. The idea conveyed in Dr. Smith's (H. A.) paper where the pulp dies and putrefies with no external entrance for germs (except through the circulation) is very interesting. In many cases, death of the pulp from accident or blow, the portion in the canal is the last to die, the body itself dying first; this is a good instance of the germs gaining access through the circulation.

If germs could get in from the gingival margin, then we would expect to find the putrefaction beginning at the apex.

*Dr. M. H. Fletcher.*—I wish to say that in my experience in inflammation of the periosteum relief can be obtained by applying the positive pole of the interrupted electric current to the affected part for half an hour. I have found that (in an experiment upon the bladder of a living frog) the electric current will produce a resumption of the circulation after stasis has taken place.

*Dr. H. A. Smith.*—I am not disputing the ability of hydrogen peroxide to destroy germs. The bi-chloride of mercury stands at the head of the list of germicides and I think it is preposterous to say that cure after cure can be performed by hydrogen peroxide without a recurrence, as it is entirely too soon for us to make such a statement as the list of germicides is constantly being augmented, each new one being proclaimed as the *ne plus ultra.*

*Dr. Fletcher.*—I saw a statement in some journal that germs have been found growing in carbolic acid itself.

*Dr. Smith.*—Some have advocated the use of a mixture of carbolic acid and glycerine; if the acid itself is no true germicide, then a solution or mixture of it would necessarily be of less value.

*Dr. G. W. Smith.*—I keep a full labeled account of all I do to a tooth, and I find hydrogen peroxide is as good or better than anything I have ever used and besides is most gentle.

*Dr. Sage.*—Dr. Smith (G. W.) does not reach the point that Dr. Smith (H. A.) raised in his paper.

*Dr. G. W. Smith.*—You forget that Dr. Smith said abscesses could be cured by injections of tepid water as well as by injections of peroxide of hydrogen.

*Dr. Sage.*—The remedy does not appear to me to be all that Dr. Smith (G. W.) claims for it, and besides it is no new remedy.

*Dr. H. A. Smith.*—One of the students in the college had a suppurative abscess incident upon the death of the pulp; it was treated and treated with peroxide of hydrogen but it did not cure the abscess.

*Dr. J. I. Taylor.*—Why didn't you try water?

*Dr. Smith.*—It is just as good.

*Dr. G. W. Smith.*—Did you try carbolic acid?

*Dr. H. A. Smith.*—Yes, we tried all the known remedies.

*Dr. O. A. Rawls.*—The burden of the discussion has tended to the effects of micro-organisms on a tooth with its pulp dying or dead. While this is one phase of the question, it is by no means *the* phase that should engage our consideration. The papers read were opposed to each other in this, that the germs can be found within a closed cavity, so far as we know, and we have evidence that putrefactive processes take place. One gentleman claims that Per Oxide of Hydrogen is not a true germicide; I apprehend that it causes a solution of pus, or dead pus, so that it can be wept out by an effervescence of the gas it gives off.

As to the possibility of germs being found within a closed cavity I do not think it is so, for the reason that if you cut off a nutrient current, you form a nidus that gives a harbor for their formation; that I do not believe.

Why is it that you have the evidences of putrefaction in a pulp unexposed to the air, smell of gas, etc? Is it that the germs get in through the medium of the circulation? No! I think it is because oxygen gets in through the tissues of the tooth.

We do not recognize the true underlying principle. We cannot produce a perfect cure by the use of any specific remedy. We can only remove, so far as we know, all the decomposing and pertrescent matter; and then what have you? A dead tissue on the inside, and a half living tissue on the outside, which opposes the only barrier between life and death. Your cure will come no matter what you use, so that you prevent the access of oxygen to set up the putrefying process in the tissue you seek to protect. When the current of nutrition, arterial or venous, between the outside and inside, has been cut off, you cannot have other than a dead tissue, and the tooth is preserved only by the integrity of its own tissue, by toleration of the powerful system of the patent in whose mouth the tooth is." Subject passed.

Dr. C. M. Wright then read a paper upon the sixth subject of the programme, followed by Dr. Watt, who read a paper on "Probable or Possible Metallic Poisoning," written by a physician, both of which are printed elsewhere in the REGISTER.

*Dr. Sage.*—This is a subject that ought to interest every dentist. The use of amalgam is called in question on account of the harm that is said to result. I have often asked dentists if they have ever seen undoubted cases of ptyalism arising from the use of amalgam, and have never received an affirmative answer.

I have myself seen but one case. In another instance I removed six or eight large amalgam fillings from the teeth of a young lady at her own request.

*Dr. H. J. McKellops.*—What was the condition of the tooth substance?

*Dr. Sage.*—Black, as it usually is under such circumstances, there was no pain no periostitis, the lady complained of an uncomfortable sensation in the mouth; she wore a partial gold plate.

*Dr. Osmond.*—Did the plate come in contact with the fillings at any point?

*Dr. Sage.*—No; the fillings were all in the lower teeth, while the plate was an upper piece.

*Dr. McKellops.*—There is no doubt of the truth in the paper just read by Dr. Watt. Any one who has removed amalgam fillings will find it very difficult to replace them with gold for the reason that the tooth-substance has become so disintegrated that it fritters away under the instrument.

The amalgam does harm in opening the door to those who are both lazy and incompetent, and besides when one is tired, he is often sorely tempted to neglect the proper performance of an operation by resorting to the amalgam.

*Dr. Osmond.*—To begin with there are many teeth that cannot be filled with gold. What I mean by amalgam is not a surplus lot of mercury but an amalgam properly made.

*Dr. Berry.*—I have seen cases where shocks of pain were produced by two fillings coming together, one of amalgam and the other of gold. The galvanic currents induced in the fillings of amalgam produce decay and so cause the fillings to fail.

*Dr. Leslie.*—Mercury evaporates at all temperatures as can be demonstrated by suspending a piece of gold foil in a bottle over the surface of mercury; after a time the surface of the gold will be found covered with it.

*Dr. Watt.*—The paper made the point of the probable or possible poisoning from the use of dentist's amalgams.

My own opinion is that in Ohio, 20,000 people suffer so much from atmospheric changes that they may be classed as invalids, and this is solely for the reason that they have amalgam fillings in their mouths. The subject is one that should be studied and considered by physicians, though I don't think there are fifty who give it any thought.

Adjourned.

SECOND DAY--MORNING SESSION.

The sixth subject of the programme was passed and the fifth taken up, Dr. J. W. Jay reading an interesting report of a case which appears upon another page of the REGISTER.

*Dr. McKellops.*—I read of an operation a few days ago which was performed by Sir Wm. McCormack, Secretary of the International Medical Congress at London. A dentist attempted to extract a tooth with a bicuspid forcep, one beak of which broke off and lodged in the trachea. Several attempts were made to disloge it, without success, a magnet having been tried in vain. It was finally removed from a point five inches below where it first lodged.

*Dr. Berry.*—An amusing case is the following: A German woman having some slight trouble with her stomach, vomited, and in doing so, spat out a partial denture she wore. A little bull pup which happened by, made a dash at the sputa and swallowed the teeth. The woman had the dog tied up, and watched him for a week, and when the teeth made their appearance, washed them off and replaced them in her mouth. (Laughter.)

*Dr. H. A. Smith.*--Great advance has been made in abdominal surgery of late years, though the trouble in these cases of removing foreign bodies from the intestines, is to locate it. The operation can be successfully performed, using the Lister method and drawing the cut surfaces together with sutures."

Subject passed.

The third subject was then taken up, Dr. J. Taft reading a paper prepared by Dr. Low, of Chicago.

*Dr. G. W. Keely.*—I would like to ask those present, if any of them remember of ever having made bridge-work, many years ago?

On this question of Bridge Work, it seems that an old thing has become new, for the reason the same principle was used many years ago. When I was a pupil of Dr. John Allen, of this city, (but now of New York,) in 1842, it was a common practice of his to insert any number of the superior teeth, on a very narrow gold plate, securing them to two or more healthy roots—roots being prepared as for a Pivot tooth. A gold pin was soldered to the plate so as to fit in the center of hole in roots. Then wooden bushes made from well-seasoned hickory, was fitted to gold pins and roots, then all pressed up to place. It is astonishing, how some such operations lasted, giving substantial service for many years. In 1844, I inserted for a lady, eight superior teeth on this principle, fastening them to the roots of the cuspids, and one central, running clasp around the second bicuspids. In 1875, I removed the plate and roots, the cuspids were quite firm, though the gums had receded from them, and the whole apparatus looked seedy, the second bicuspids and molars were all broken down, and a delightful odor was there. I mention this case because it is rare that such operations last more than ten to fifteen years at best. At this time Stockton's teeth were used.

Four years ago a friend gave me a plate of this kind with six teeth on it, with small clasps that wind round the first biscuspus, and gold pins over cuspids. The teeth were evidently of some of the first porcelain teeth made in France, the body and enamel was peculiar without natural appearance. It was found when removing the remains from a grave, the subject having been buried over fifty years ago, and it is supposed the work was done in New York. This relic has been missing for about a year, it was either misplaced or purloined.

*Dr. McKellops.*—Was that case ever published ?

*Dr. Keely.*—No, but it ought to have been.

*Dr. Wm. Van Antwerp.*—Prof. Harry Smith taught that right here in this room, of course, without embellishments, but the principle was the same as that of the new methods.

*Dr. Berry.*—Many of these methods were practiced many years ago, but *now* they are forgotten and are antique. Time was that it was thought unprofessional to patent anything, but in my opinion, if a man works out anything of use, I have no objection to his taking out a patent for it and reaping all the benefit he can.

*Dr. J. P. Ullery.*—I have done bridge-work over forty years ago, and after considerable experience with it, I abandoned it as useless. My method was to solder porcelain teeth together with a bridge, and then soldered this to a band which was fastened to remaining teeth or roots.

*Dr. McKellops.*—Did I understand you to say you soldred the bridge to the *band* around the tooth and not a clasp merely?

*Dr. Ullery.*—Yes, I did.

Adjourned.

AFTERNOON SESSION.

This session was devoted to the exhibition of appliances by Drs. McKellops and W. S. How, mention of which will be found in "Salmagundi." After the payment of bills, appropriations, etc., the society elected its officers for the ensuing year and adjourned to meet the first Wednesday of March, 1887.

